# Evaluation of occlusal bite force distribution by T-Scan in orthodontic patients with different occlusal characteristics: a cross sectional-observational study

**DOI:** 10.1186/s12903-023-03544-4

**Published:** 2023-11-20

**Authors:** Huda Abutayyem, Lovely M Annamma, Vijay B Desai, Mohammad Khursheed Alam

**Affiliations:** 1 Department of Clinical Sciences, College of Dentistry, Ajman University and Center of Medical and Bioallied Health Sciences, Ajman University, Ajman, United Arab Emirates; 2 Orthodontic Division, Preventive Dentistry Department, Jouf University, Sakaka, Aljouf, Saudi Arabia; 3 Department of Dental Research Cell, Saveetha Institute of Medical and Technical Sciences, Saveetha Dental College and Hospitals, Chennai, 600077 India; 4 Department of Public Health, Faculty of Allied Health Sciences, Daffodil International University, Dhaka, 1207 Bangladesh

**Keywords:** Occlusal Bite, Force Distribution, T-Scan, Orthodontic Patients

## Abstract

**Background:**

The aim of orthodontic treatment, apart from esthetic and functional corrections, is uniform force distribution. Hence Occlusal analysis using a T scan gives scope for a precisely targeted treatment plan. The T-scan evaluation of occlusal force, time, and location of contacts from initial occlusal contact to maximum intercuspation enables the orthodontist to sequentially balance the occlusal forces on the right and left sides through specific treatment plan options.

**Objective:**

The current study aimed to determine the force distribution in the different individuals by using a T-Scan as well as the net discrepancies of forces generated at a maximum intercuspation position in the first molar region between the left and right sides of the mouth.

**Methods:**

This is a descriptive-correlational study that was carried out in Ras Al Khaimah College of Dental Sciences clinics and Ajman University clinics from January 2020 to September 2022 by using the convenience sampling technique. The T-scan III Novus was employed in this investigation to record multi-bite scans for several patients. T-scan was utilised to examine various malocclusions.

**Results:**

The present study consisted of 158 participants. Analysis of Variance (ANOVA) showed that there is a statistically significant difference in the percentage of force between the three types of malocclusions (I, II, and III) on the right molar side (B-16 and B-46) (p < 0.05). Moreover, the overall discrepancy showed a statistically significant difference in the three types of malocclusion classifications (p < 0.05). On the other hand, there was no statistically significant difference in the percentage of force between B-26 and B-36 (p > 0.05). Post hoc analysis showed a statistically significant difference in the percentage of force between malocclusion classes I and III on the right molar, with a mean difference of 4.11190 (p < 0.05). Similarly, there was a statistically significant difference in B-46 between Malocclusion Classes I and II, 4.01806 (p < 0.05). Additionally, post hoc analysis showed a statistically significant difference between malocclusion classes I and III, with a mean difference of -4.79841 (p < 0.05) on the right molar.

**Conclusion:**

The T-Scan is a useful tool for assessing occlusal discrepancies and can be helpful during treatment planning and follow-up, especially for orthognathic surgery patients. A T-scan could be used in orthodontic therapy in a simple and efficient way. Also, it turned out to be a useful tool for diagnosing problems and gave us new information about how therapies work. In this study, T-Scan showed that it can measure occlusal forces in timing in an objective, accurate, and repeated manner. The current study found that T-Scan was better able to report the difference in the percentage of force on the right molar side than on the left side.

## Introduction

Occlusal force represents the function of mastication. Using measurable occlusal indicators provides precise occlusal force evaluation. In modern dental clinics, digital methods such as T-scan are frequently used to assess treatment outcomes. The T-scan reveals the relative bite force (percent) relative to the maximal bite force on individual teeth or the unilateral arch. It is challenging to find a device that can satisfy all requirements for recording occlusal [1].

The contact between the upper and lower dentition while the teeth are in maximum intercuspation is defined as dental occlusion [2]. Furthermore, the phrase “dynamic occlusion” refers to tooth interactions that occur during mandibular movements [3]. Because the number, location, and position of the teeth vary so significantly, the conceivable combinations of distinct forms of dental occlusion are enormous. As a result, numerous traits have been classified to describe categories of malocclusions in order to investigate dental occlusion. Angle’s classification, which differentiates distinct types of dental occlusion based on the sagittal relationship between the upper and lower teeth in orthodontic patients is a widely used classification [4]. The orthodontic therapeutic goal is to achieve an ideal alignment of teeth in the dental arch embracing static occlusion that permits an even distribution of the generated forces during mastication. For instance, any premature occlusal contact can generate occlusal stress which leads to alterations in the tooth-supporting tissues, the masticatory muscles, and the temporomandibular joint [5].

Following orthodontic treatment, the retention phase is designed to keep the correct occlusion and function. Without retention, recurrence or an adverse change from the ultimate occlusion is expected [6]. Stabilization is a positive improvement that occurs following orthodontic treatment. Teeth will naturally erupt toward one other in search of stable sites of contact, enhancing intercuspation and masticatory performance. The number of occlusal contacts rises with settling [7]. Since the introduction of modern orthodontics, dental occlusion, and occlusal pressures have been proposed as one element for stability [8].

**Fig. 1:**
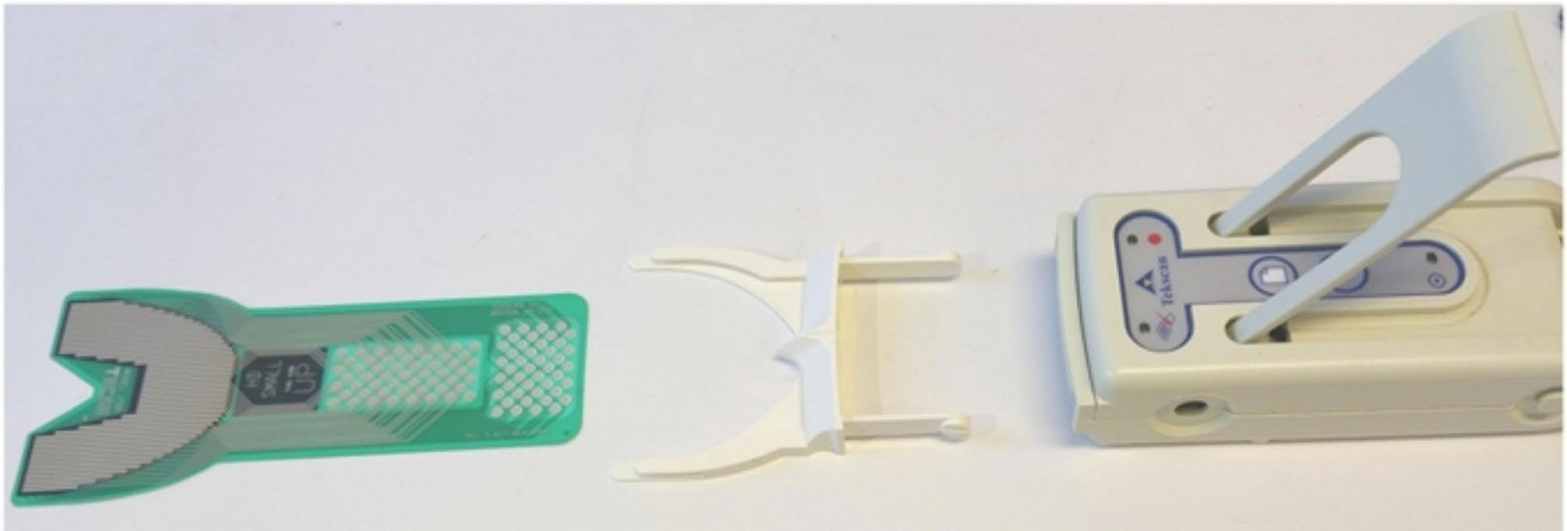
T-Scan system

The T-Scan is an objective approach for assessing dynamic dental occlusion. It enables computerized analysis, which eliminates operator subjective paper mark misperceptions; additionally, T-Scan measurements are unaffected by saliva [9]. T-Scan is a digital occlusion analysis device that uses a small, flexible, pressure-sensitive bite transducer inserted in a dental arch-shaped recording sensor to record and evaluate tooth contact, force, and timing in real-time [10]. T-Scan occlusal data can be graphically displayed for study in two or three dimensions (Fig. 1). The recorded occlusal data can be used to calculate the occlusal force distribution, occlusal interference, and relative force of each interference. The T-Scan records patient parameters such as the center of force, confirming the occlusal force’s symmetry. It can determine the first contact between maxillary and mandibular teeth, the maximum biting force, the maximum intercuspation, and the occlusal position of the mandible in which the cusps of the maxillary teeth fully interpose with the cusps of the opposing arch. Maximum intercuspation is a crucial jaw position that defines the mandibular and maxillary anterior-posterior and lateral relationships, as well as the superior-inferior relationship known as the vertical dimension of occlusion. When evaluating an orthodontic patient, maximum intercuspation is critical [8]. By translating qualitative data into quantitative parameters, the T-scan 10 system provides a precise means of assessing the sequence of time and occlusal contact force magnitude. By displaying it on a digital display, it also boosts the patient’s confidence [11].

To the best of the authors’ knowledge, there are no studies investigating the occlusal bite force distribution by T-Scan in orthodontic patients with different occlusal characteristics in the United Arab Emirates. Therefore, the goal of the current study is to analyze bite force distribution in patients with different occlusal characteristics by using a T-scan. Moreover, the secondary objective is to determine the net discrepancies of forces generated at a maximum intercuspation position in the first molar region between the left and right sides of the mouth. To achieve this, the objectives were set to utilize T-Scan technology as a precise tool for quantifying and visualizing bite forces among orthodontic patients. Additionally, the study aimed to provide orthodontists and researchers with valuable insights into how these occlusal characteristics might impact treatment planning, appliance design, and post-treatment stability.

## Methodology

### Design, setting, and sampling

This is a descriptive-correlational study that was carried out by a single examiner in Ras Al Khaimah College of Dental Sciences clinics and Ajman University clinics from January 2020 to September 2022 by using the convenience sampling technique.

### Inclusion and exclusion criteria

The following inclusion criteria were taken into consideration during participant selection: class 1 facial profile and standard facial height with no history of orthodontic treatment; no missing teeth in the molar region; no pain related to the molars; no heavily restored teeth in the molar area; no gingival inflammation, periodontal pathology, or absence of tooth mobility; and no reported systemic disease (chronic arthritis) or apparent facial asymmetry that could be corrected by orthodontics. On the other hand, participants with TMJ disorders and patients with other systemic, congenital, and traumatic disorders affecting the jaw, chronic periodontal disease, and any missing tooth apart from third molars were excluded from this study.

The absence of an intervention group did not hamper the objectives of our investigation since the primary focus was on assessing the relationships between various occlusal characteristics and bite force distribution patterns among orthodontic patients. Unlike experimental or interventional studies, where interventions are intentionally applied to a group to observe their effects, cross-sectional observational studies are designed differently.

In this study, data were collected from orthodontic patients with varying occlusal characteristics, including malocclusions, dental arch shapes, and tooth misalignments. The absence of an intervention group did not hinder the study’s aims because the research design was not intended to assess the effects of an intervention. Instead, it aimed to establish correlations and associations between these occlusal traits and the distribution of bite forces during mastication.

### Sample size

To achieve a power of 0.80, the power analysis was performed by using the G*Power3 software, with the alpha level set to 0.05 and the medium effect size (d = 0.30) [11]. Therefore, the estimated sample size of 128 patients has an 80% probability of detecting a true difference (of medium effect size) between the four groups when the significance level is set at p < 0.05.

### Study procedure

The research was reviewed and approved by the Research Ethics Committee (Ref# D-H-F-11-Nov). Patients seeking orthodontic treatment at Ras Al Khaimah College of Dental Sciences and Ajman University Clinics were asked to voluntarily participate in this study. The study protocol was explained to each potential subject, and signed consent was obtained for those who agreed to participate and fulfilled the following inclusion criteria: complete permanent teeth excluding the third molars, normal temporomandibular joint function, and absence of periodontal pathology. Patients were invited to be seated on the dental chair with the lower and upper parts of their body positioned at an angle of 90o.

**Fig. 2:**
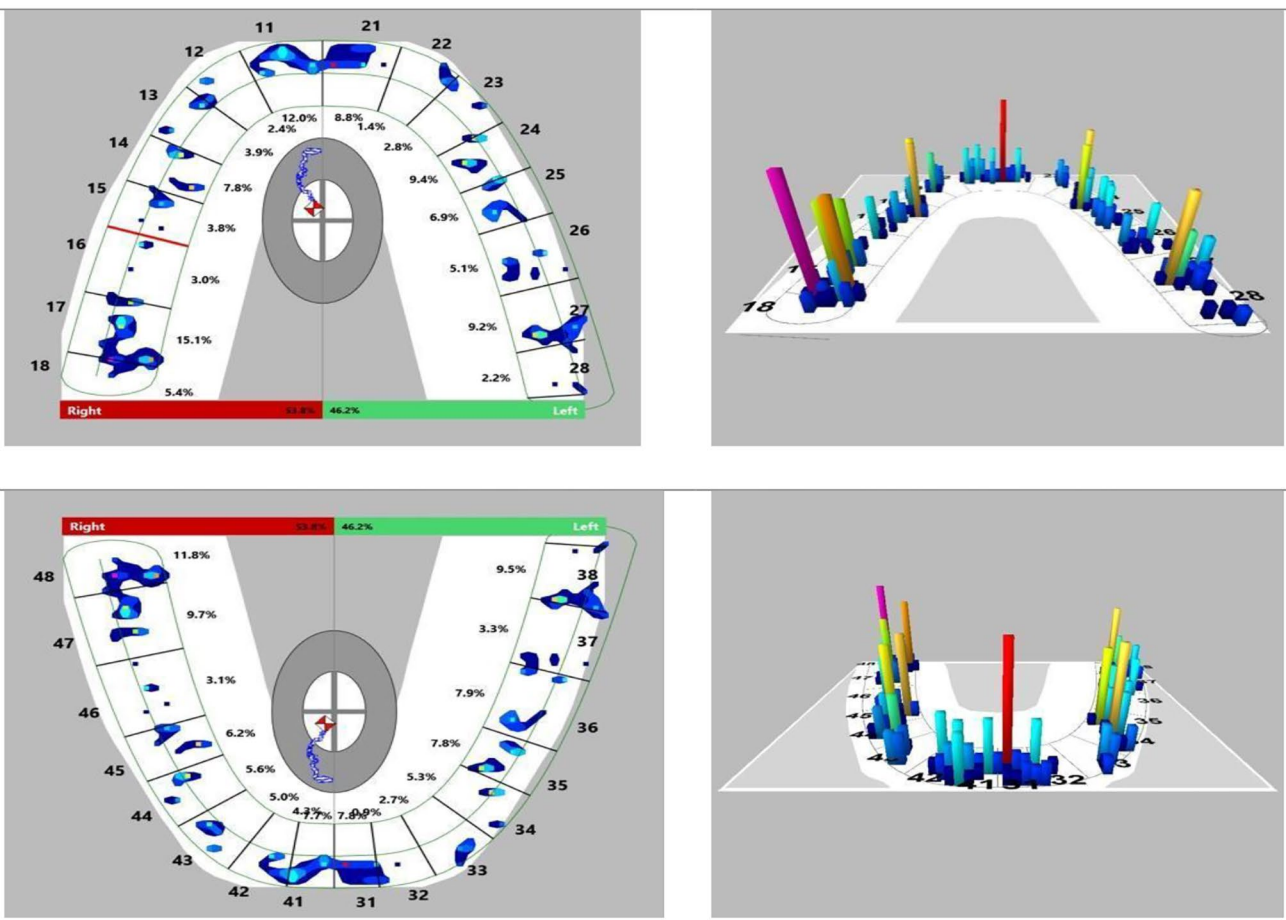
-2-D and 3-D Images of Occlusal contacts and force generated

**Fig. 3:**
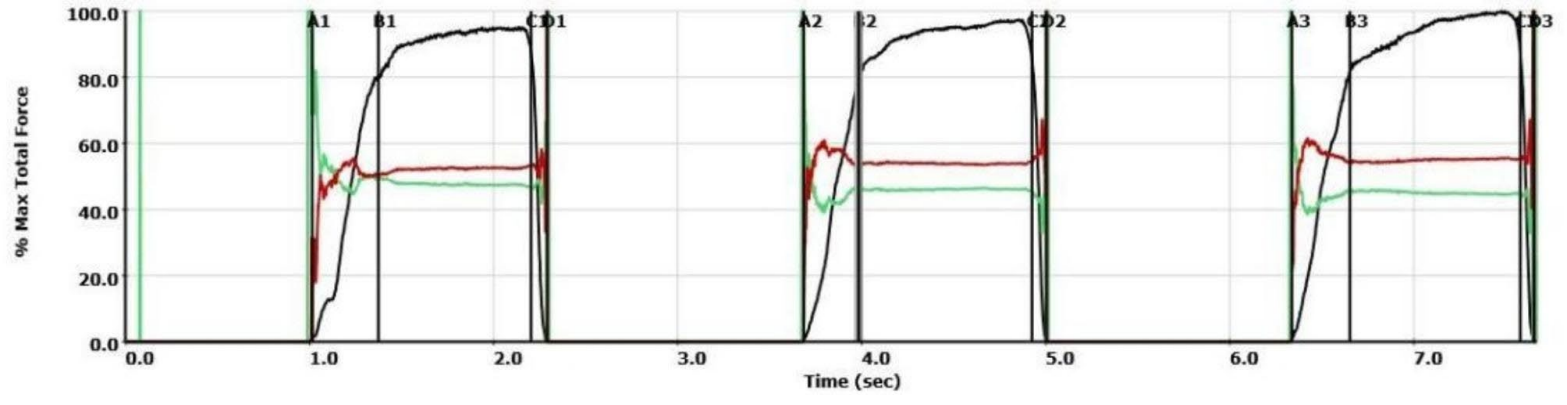
Graph on Maximum force generated with time on occlusal contact

In our study, the T-scan III Novus was used to record multi-bite scans for multiple patients. T-scan was used to study different types of malocclusions; Angle’s Class 1 malocclusion, Angle’s Class II malocclusion, Angle’s Class III malocclusion, and normal occlusion. The procedure was carried out by using the T-Scan III NOVUS device that consists of a sensor film registering occlusal contacts, a data transferring module linked the sensor to a computer called the ‘handpiece’. A software program collects the gathered data and transfers it to the computer enabling visualizing the captured data in 2 and 3-D formats on the monitor. The recording sensor is inserted intraorally between the dental arches so that the central mark is positioned between the central incisors of the patient. Recording starts with pressing the button on the handlebar; the patient is instructed to occlude firmly to complete intercuspation. A multi-bite type scan was recorded for each subject consisting of 3 bites consequently after each other to minimize patient error. One of the key features provided by the T-SCAN software is a force vs. time graph. On each graph deduced, 4 dimensions are written by the software. These dimensions are marked by points on the graph. Points A, B, C, & D (Fig. 2). Point A represents the first contact point that occurs upon occluding. Point B represents the maximum intercuspation position (MIP) when the patient is in 100% full occlusion. Point C symbolizes the first disclosure between the teeth that occurs upon releasing the occlusion load. Finally, point D represents full disclosure where no teeth are expected to be in contact. (Fig. 3).

The average value in these 3 bites was the readings taken into consideration in this study. The B point interval which represents the MIP (Maximum intercuspal position) was the dimension of interest in this study. Another method of data provided by the software is the occlusal load percentage bared by each tooth alone. This is given in either a 2D image or a 3D image according to preference.

The occlusal forces were evaluated for each dental element, as well as for each side (left and right) and sector (back and front), using identical methods for each individual studied. The inter-incisive line on the occlusal axis demonstrated that the right and left sides were separate.

After extracting the reports for all studied specimens from the software, the average bilateral occlusal load was calculated for each specimen in each class by adding all occlusal forces on the right side and left side of the jaws. The net discrepancies were deduced by subtracting those two numbers to find the differences in the forces distributed along both sides of the jaws. Furthermore, to find the total average occlusal force of the first molar in each class in MIP, the values of the four first molars in each specimen were compacted into one value which was the average of the four molars altogether. From that value, the classes were compared with each other using multiple statistical tests.

### Calibration procedure

Fastier [12] proposed a low-cost, dependable sensor for measuring the maximum voluntary bite force. Two polyvinylsiloxane (PVS) silicone layers, an acrylic frame, and a metal strain gauge compose the sensor. The PVS silicone resin functions as a protective layer to lessen the discomfort the subject may experience when biting the sensor. A strain gauge attached to the interior of the acrylic resin functions as the sensing element, and an acrylic frame transmits the mechanical strain caused by the bite force to the strain gauge. The sensor is created for simple fabrication, assembly, calibration, and use. It can be utilized one hour after production begins, allowing for rapid prototyping and modification. The data indicates a linear relationship between the applied force and sensor resistance [12].

### Statistical analysis

The statistical analysis was conducted using the Statistical Package for Social Sciences version 26 (SPSS; IBM Corporation, Armonk, NY, USA). The Shapiro-Wilk test indicated that the data were approximately normally distributed. ANOVA was performed to evaluate the association between three types of teeth classification. Post hoc multiple comparisons were performed using the Tukey-HSD method to detect significant intergroup differences. The significance level was set at p < 0.05.

## Results

The present study consisted of 158 participants. The majority were males 99 (62.7%) and females 59 (37.3%). In terms of nationality, 90 (57%) were UAE citizens, followed by Indians 27 (17.1%), Pakistanis 18 (11.4%), and 23 (14.6%) from different nationalities. The data was collected from two universities, 85 (53.8%) from Ras Al Khaimah College of Dental Sciences 73 (46.2%) and from Ajman University. In regard to the malocclusion classification, 84 (53.2%) had Class I Malocclusion, 32 (20.3%) had Class II Malocclusion and 42 (26.6%) had Class III Malocclusion. The mean age of participants was 28.27 ± 11.31. (Table 1).

**Table 1:** Characteristics of participants

Variable	Group	Frequency		Percentage	Mean and SD
Gender	Males	99		62.7	28.5 ± 4.2
	Females	59		37.3	30.2 ± 3.8
Nationality	UAE Citizen	90		57.0	29.8 ± 4.0
	Pakistani	18		11.4	27.3 ± 3.5
	Indian	27		17.1	28.7 ± 3.9
	Others	23		14.6	31.0 ± 4.3
Site of data collection	Ras Al Khaimah college of dental	85		53.8	29.4 ± 4.1
	Ajman University	73		46.2	30.1 ± 3.7
Malocclusion classification	Malocclusion Class I	84		53.2	30.5 ± 5.1
	Malocclusion Class II	32		20.3	27.8 ± 4.3
	Malocclusion Class III	42		26.6	32.2 ± 5.7
**Continuous variables**					
	**N**	**Minimum**	**Maximum**	**Mean ± S. D**	
Age	158	15.00	64.00	28.27 ± 11.31	

Analysis of Variance (ANOVA) was performed to find the mean differences between three types of teeth classification for each B-16, B-26, B-36, and B-46. The result showed that there is a statistically significant difference in the percentage of force between the three types of malocclusions (I, II, and III) on the right molar side (B-16 and B-46) (p < 0.05). Moreover, the overall discrepancy showed a statistically significant difference in the three types of malocclusion classifications (p < 0.05). On the other hand, there was no statistically significant difference in the percentage of force between B-26 and B-36 (p > 0.05). (Table 2).

**Table 2:** Analysis of Variance (ANOVA) result of tooth malocclusion classification

		Sum of Squares	df	Mean Square	F	Sig.
B 16	Between Groups	713.518	2	356.759	7.114	0.001
	Within Groups	7773.541	155	50.152		
	Total	8487.059	157			
B 26	Between Groups	39.344	2	19.672	0.304	0.738
	Within Groups	10029.258	155	64.705		
	Total	10068.601	157			
B 36	Between Groups	36.708	2	18.354	0.300	0.741
	Within Groups	9472.450	155	61.113		
	Total	9509.159	157			
B 46	Between Groups	793.968	2	396.984	20.648	0.000
	Within Groups	2980.136	155	19.227		
	Total	3774.104	157			
Overall Discrepancy	Between Groups	17651.771	2	8825.886	205.455	0.000
	Within Groups	6658.445	155	42.958		
	Total	24310.216	157			

Tukey-HSD test was used to detect significant intergroup differences. There was a statistically significant difference in B-16 between Malocclusion Classes I and II, with a mean difference of 4.43800 (p < 0.05). Additionally, post hoc showed a statistically significant difference between Malocclusion Class I and Class III with a mean difference of 4.11190 (p < 0.05). (Table 3).

**Table 3:** Multiple Comparison (Tukey HSD) of **B-16** between 3 types of tooth malocclusion classification

Dependent Variable: B 16						
Tukey HSD						
(I) Class	(J) Class	Mean Difference (I-J)	Std. Error	95% Confidence Interval		Sig.
				Lower Bound	Upper Bound	
Malocclusion Class 1 (B-16)	Class I (B16)	4.43800^*^	1.47115	0.9566	7.9194	0.008
	Class III (B16)	4.11190^*^	1.33833	0.9448	7.2790	0.007
Malocclusion Class II (B-16)	Class I (B16)	-4.43800^*^	1.47115	-7.9194	− 0.9566	0.008
	Class III (B16)	− 0.32609	1.66173	-4.2585	3.6063	0.979
Malocclusion Class III (B-16)	Class I (B16)	-4.11190^*^	1.33833	-7.2790	− 0.9448	0.007
	Class II (B16)	0.32609	1.66173	-3.6063	4.2585	0.979

*. The mean difference is significant at the 0.05 level

Also, there was a statistically significant difference in B-46 between Malocclusion Classes 1 and 2, with a mean difference of -4.01806 (p < 0.05). Additionally, post hoc showed a statistically significant difference between Malocclusion Class 1 and Class 3 with a mean difference of -4.79841 (p < 0.05). (Table 4).

**Table 4:** Multiple Comparison (Tukey HSD) of **B-46** between 3 types of tooth malocclusion classification

Dependent Variable: B 46						
Tukey HSD						
(I) Class	(J) Class	Mean Difference (I-J)	Std. Error	95% Confidence Interval		Sig.
				Lower Bound	Upper Bound	
Malocclusion Class I (B-46)	Class II (B-46)	-4.01806^*^	0.91089	-6.1736	-1.8625	0.000
	Class III (B-46)	-4.79841^*^	0.82865	-6.7594	-2.8374	0.000
Malocclusion Class II (B-46)	Class I (B-46)	4.01806^*^	0.91089	1.8625	6.1736	0.000
	Class III (B-46)	− 0.78036	1.02889	-3.2152	1.6545	0.729
Malocclusion Class III (B-46)	Class I (B-46)	4.79841^*^	0.82865	2.8374	6.7594	0.000
	Class II (B-46)	0.78036	1.02889	-1.6545	3.2152	0.729

*. The mean difference is significant at the 0.05 level

The mean of three types of tooth malocclusion classification was calculated to find the difference in net discrepancy. The post hoc analysis showed a statistically significant difference between the net discrepancy of Malocclusion Class 1 and the net discrepancy of Malocclusion Class II, with a mean difference of -26.20 (p < 0.05), but not with the net discrepancy of Malocclusion Class III (p > 0.05). Furthermore, the result showed a statistically significant difference between the net Discrepancy of Malocclusion Class II the and Net Discrepancy of Malocclusion Class III, with a mean difference of 26.49320 (p < 0.05). (Table 5).

**Table 5:** Multiple Comparison (Tukey HSD) of Net Discrepancy between 3 types of tooth malocclusion classification

Dependent Variable: Net Discrepancy						
Tukey HSD						
(I) Class	(J) Class	Mean Difference (I-J)	Std. Error	95% Confidence Interval		Sig.
				Lower Bound	Upper Bound	
Net Discrepancy of Malocclusion Class 1	Class II	-26.20134^*^	1.36155	-29.4234	-22.9793	0.000
	Class III	0.29187	1.23863	-2.6393	3.2230	0.970
Net Discrepancy of Malocclusion Class II	Class I	26.20134^*^	1.36155	22.9793	29.4234	0.000
	Class III	26.49320^*^	1.53793	22.8538	30.1326	0.000
Net Discrepancy of Malocclusion Class III	Class I	− 0.29187	1.23863	-3.2230	2.6393	0.970
	Class II	-26.49320^*^	1.53793	-30.1326	-22.8538	0.000

*. The mean difference is significant at the 0.05 level

## Discussion

The current study aimed to analyze bite force distribution in patients with different occlusal characteristics by using a T-scan. Moreover, the secondary objective is to determine the net discrepancies of forces generated at a maximum intercuspation position in the first molar region between the left and right sides of the mouth. Malocclusion is the third major oral health problem, which may affect self-esteem due to aesthetic, speech, functional, and psychosocial changes, impairing the individual’s quality of life [13]. Thus, appropriate indices for the analysis of malocclusions in population studies should be developed, emphasizing their functionality in determining the need and priority for treatment in addition to detecting objective signs and providing information that allows for careful social analysis and the rational allocation of human, material, and financial resources for orthodontic therapy in public health [14]. Occlusal bite force indicates functional mastication and tooth loading, which results in jaw elevations using muscles determined by the central nervous system and retrogressed from muscle spindles, mechanoreceptors, and nociceptors, modifying craniomandibular biomechanics. A stronger bite force results from a superior masticatory mechanism [15]. According to one study, bite force levels are employed to investigate mastication mechanics and therapeutic outcomes [16].

Wang’s study showed that the T-Scan system’s recordings are clinically useful in terms of accuracy and repeatability when looking at occlusal contact in the lateral excursion [17]. Saliva in the mouth doesn’t change the way the T-Scan system records [18]. In the same way, other clinical and laboratory research has confirmed the T-Scan system’s pressure sensitivity, accuracy, and stability of relative force loadings, as well as the repeatability of results [19].

The T-Scan, which can detect unequal distribution or relative occlusion, will highlight where excessive force is concentrated, and variations in occlusion over time will be more therapeutically useful than measuring absolute occlusion force because it can be misleading [20]. The T-scan system’s advantages include not only its objectivity and reproducibility but also its ability to identify occlusal changes over time. This system measured parameters that time-related factors, occlusal papers, and occlusal indices could not. Furthermore, this method is currently the only one accessible for investigating the dynamic properties of occlusion [21].

The current study found a difference in the percentage of force in the right upper molar (B-16) between malocclusion classes 1 and 2, as well as classes I and III. Similarly, the percentage of force in the right upper molar (B-46) differed statistically between malocclusion classes 1 and 2. However, no difference in percentage of force was observed in any of the malocclusion classifications observed in (B-26 and B-36) classes I, II, or III.

T -Scan ability to report the difference in percentage of force on the right molar side was superior to the left side. The T-Scan occlusal pattern did not correspond with the malocclusion’s angle categorization. Similarly, Agbaje found that T-scan could not detect class II or class III malocclusions depending on the position of the teeth on the arch and relative to the opposing jaw [8]. Furthermore, Alhammadi discovered that the T-scan is less effective at detecting occlusal function patterns in patients with severe skeletal class III and skeletal class II malocclusion. Except for teeth 46, 44, and 41 [20]. González reported no significant changes in the proportion of force on each tooth following four bites done in a maximal intercuspation position using a T-Scan [21]. However, significant scientific data supports the use of the T-Scan since it assesses relative occlusal forces and time objectively, correctly, and repeatedly. The computerized occlusal analysis method has been extensively researched and may offer exact time and force sequencing information to objectively evaluate occlusal contacts for better treatment outcomes [22]. Other research suggests that several parameters, such as the chewing side and the inactivity of the other side of the jaw, alter the accuracy of T-scan findings [23]. The expression of the higher muscle force on the preferred chewing side is connected with the higher occlusal force on that side. Lower cervical muscle activity has been linked to a decreased occlusal contact area [24]. Lower force applied on a non-preferred chewing side is related to a “weaker” chewing muscle, and therefore with a smaller occlusal contact area, whereas the smaller occlusal contact area is connected with a reduced occlusal force [25].

The current study found a statistically significant difference in the net discrepancy of Malocclusion Class I and Class II (p < 0.001), as well as the net discrepancy of Malocclusion Class II and Class II (p0.001). Age, gender, skeletal morphology, and malocclusion could all affect the net disparity of malocclusions, and the first molars are subjected to the most stress during chewing [22]. A study compared the occlusal strength parameters in 25 individuals with Angle class I, II, and III relationships with and without orthodontic treatment using T-Scan III, and the largest amount of force was concentrated on the second molars in both groups, followed by the first molars and second premolars, respectively. According to the study, the lateral incisors were subjected to less force. The study also indicated that the distribution of stresses on the teeth inside the arch varied between 0% and 35% [22]. In contrast, another study discovered no significant variation in force per tooth between the three categories of malocclusion classification I, II, and II. Furthermore, the occlusal forces were distributed evenly in the right and left jaws, and there was no significant difference in the occlusal force distribution in the right and left jaws [26]. Another study found that the occlusal forces in the right and left hemispheres had a balanced distribution that did not exceed 50% on one side [9]. Furthermore, it has been noted that the percentage of force on the non-working side observed in individuals who have undergone orthodontic treatment is similar to that of healthy individuals who have not undergone treatment, with a higher prevalence of group function occlusion pattern in the former [27]. Another study found that the increase in force distribution on the non-working side in individuals who had orthodontic treatment was induced by contacts, particularly on the second molar teeth [8].

Many studies have described the clear advantages of quantitative and qualitative T-scan processes over traditional qualitative approaches, especially because they avoid the practitioner’s subjective judgment [28]. Numerous research studies have been conducted to assess the reliability and validity of the T-Scan, indicating that it might be considered suitable for clinical application [8, 29]. Previous generations of T Scans I and II, which included significantly stiffer sensor foils, elicited opposing views on reproducibility [30]. However, Koos found no flaws in their reliability analysis of the T Scan III [29], which was verified by another study [31, 32].

## Conclusion

The obtained findings revealed significant variations in force distribution, particularly between Class I malocclusions and both Class II and Class III malocclusions. These findings carry significant clinical implications for orthodontic practice. By recognizing the distinct force patterns associated with different malocclusion classifications, orthodontists can develop more personalized treatment strategies. This individualized approach can lead to improved treatment outcomes and enhanced patient satisfaction. Furthermore, our study contributes to the growing body of knowledge in orthodontics and dental research. Understanding the intricate dynamics of occlusal bite forces is pivotal in refining orthodontic interventions and optimizing patient care. While this study sheds light on the immediate implications for orthodontic practice, it also highlights the potential for further research in this domain. Future investigations could explore additional factors influencing bite force distribution and their impact on orthodontic treatment. Overall, our findings underscore the importance of tailoring orthodontic interventions to the unique characteristics of each patient’s malocclusion, ultimately enhancing the quality of orthodontic care.

### Limitations

The limitations of the current study were acknowledged and reported. The study was conducted solely to evaluate the occlusion and develop a targeted treatment plan of evaluating the occlusion and developing a targeted treatment plan. Further studies are required to do a comparative evaluation before and after orthopedic treatment. Other limitations include accuracy in sensor calibration; hence, in our study, the calibration of the sensor was done meticulously, and if the sensor suffered damage midway through the procedure, the reading was discarded, and a new sensor was used for further readings. Interferences exceeding 0.6 mm were difficult to detect.

### Clinical implication

Different types of malocclusions are treated in orthodontics. While aligning the tooth, occlusal changes occur. These occlusal changes can cause variations in biting force and pressure on the tooth. A T-scan can accurately determine the first contact of the tooth during occlusion, the maximum biting force, and the center of force, and it can record centric and eccentric contacts. Hence, T Scan, if used correctly, can be an asset in assessing occlusal discrepancies and comparing the maximum intercuspation force on teeth after and before orthodontic alignment.

A T-scan aids as a diagnostic tool to locate occlusal discrepancies not observed by the diagnostic cast and articulating paper. The abnormal forces on a tooth can be detected before treatment, and a proper treatment plan can rectify the occlusal interference. After the orthodontic alignment, the post-treatment T-scan records can be compared to the pretreatment records. This can be saved as treatment plan records and can be verified in the long term to evaluate changes occurring after a few years of treatment.

## Data Availability

The data that support the findings of this study are not publicly available due to restricted organization rules to share the data in public. However, the dataset is available with the corresponding author but with a reasonable request.
